# Factors Associated with Consuming Junk Food among Saudi Adults in Jeddah City

**DOI:** 10.7759/cureus.2008

**Published:** 2017-12-31

**Authors:** Najlaa Mandoura, Rajaa Al-Raddadi, Ola Abdulrashid, Hassan Bin Usman Shah, Sulaiman M Kassar, Abdul Rehman Adel Hawari, Jana M Jahhaf

**Affiliations:** 1 Research Department, Directorate of Health Affairs for Public Health Division, Jeddah; 2 Consultant Preventive Medicine, Joint Program for Preventive Medicine, Jeddah; 3 Head of Research Unit, Directorate of Health Affairs for Public Health Division, Jeddah; 4 Final Year Mbbs Student, King Abdul Aziz Medical College, Jeddah

**Keywords:** adults, junk food, prevalence, primary health care centres, restaurants

## Abstract

Introduction

Junk food (JF) consumption trend is increasing in all parts of the world. The transition in lifestyle and dietary habits is leading to many non-communicable diseases. The objectives of this study are twofold: (1) To examine the prevalence of junk food consumption and factors associated with consuming junk food among Saudi adults in Jeddah; and (2) to compare the trends of junk food consumption among males and females in Jeddah.

Methodology

This cross-sectional study was conducted in five different Primary Health Care centers (PHCCs) of Jeddah working under Ministry of Health. The subjects were men (n = 146) and women (n = 254) aged 18-67 years visiting these centers. Structured validated close ended questionnaire was filled by all the participants. Data analysis was done using SPSS. Chi-square was applied to analyze the difference between male and female JF consumption and multivariate logistic regression analysis was done to examine the risk factors.

Results

Overall the JF consumption in subjects with mean age 33.69 ± 12.29 years was highly prevalent in both genders (86.5%); (men = 85.6% and women = 87.4%). Controlling for some demographic and socioeconomic variables, increased junk food consumption was independently associated with education (OR = 2.47, 95% CI: 1.088-5.605, p = 0.031), individuals who had limited time (OR = 3.82, 95% CI: 1.690-8.642, p < 0.001), for the change of routine and taste (OR = 7.64, 95% CI: 3.145-18.563, p < 0.001 and OR = 11.031, 95% CI: 4.219-28.843, p < 0.001, respectively).

Conclusion

The study findings provide evidence on the high prevalence of junk food consumption among Saudi adults. Junk food has influence in the dietary patterns of Saudi adults and this trend is likely to rise. This growing widespread use of junk food is of concern which may cause obesity-related non-communicable diseases.

## Introduction

Junk food (JF) is defined as a food which is readily available, usually inexpensive, may or may not be nutritious [1]. Such food contains more calories, more salt, have a higher content of saturated fat and contains less iron, calcium and dietary fiber [2,3]. Common junk food includes fast food, carbonated drinks, chips, desserts, chocolates, etc. [4]. Adult’s food choices are not consistent with the dietary guidelines, leading to many preventable diseases [5]. Over the past few decades, junk food consumption has increased worldwide [5]. Consuming large amounts of junk food is associated with a dramatic decrease in healthy food like milk, fruits and vegetables intake [6]. High income, rapid urbanization, free home deliveries, mouthwatering advertisements and international cuisines have contributed to a rising trend in increased junk food intake [4,7].

Visible changes in the lifestyle patterns are noticed once the individual reaches his/her adolescence [8]. The tradition of family dinner is getting replaced by eating “on the run” [9]. Unfortunately, these modifications are usually not healthy, ranging from eating junk food in restaurants to lack of physical activity [10, 11]. Based on some recent reports, more than one-third adults consume junk food two or even more times during a week [12]. Other reasons for these unhealthy habits include eating junk for pleasure, taste, laziness, friends company, independence and easy availability of these amenities [8-10]. These unhealthy habits have several adverse effects on health [11,12].

Nutritious food plays an important role in the body development and prevention of diseases. Modern day deviation from organic and pure diet affects an individual’s health [13]. Studies prove such food to cause obesity (central adiposity); a primary cause of heart diseases and other non-communicable diseases (NCDs) [2,4,14]. The burden of NCDs has become a major public health concern all over the world, attributed to the unhealthy life style which includes unhealthy dietary habits, physical inactivity and smoking [5,8,9]. Fortunately, NCDs are preventable [7]. A strong association lies between NCDs and lifestyle habits [7,9-11].

Increase in the junk food consumption is a global phenomenon having a prevalence of around 70% [13,14]. It is considered as an emerging major public health challenge among all age groups and especially in young adults with a male predominance [12,14]. Schmidt, et al. [15] highlighted that the frequency of fast food consumption increases with increase in age (from adolescent to adults). Other studies highlighting reasons for increased consumption of junk food have given an insight to avoid them, but unfortunately measures taken are not as effective as they need to be [4,6,14]. In this modern era, it seems to have engulfed members of every age and race [7,8,11].

Recent studies have shown that the trends in junk food consumption especially in the adolescents and young adults, as well as the number of fast food restaurants have significantly increased in Saudi Arabia also [8]. The dietary habits developed at a young age are important as these behaviors are likely to remain stable for the entire lifespan [14,15]. This is an important health concern especially in the urban areas. The purpose of this study is to assess the trends of junk food consumption and factors leading to its increased uptake in adults. Determining the factors for this increased JF consumption could be used as a guide for conducting interventions, aiming to help them adopt healthy eating behaviors.

## Materials and methods

A cross-sectional interview-based study with a duration of five months from 1st February 2017 to 1st July 2017 was conducted in the primary health care centers (PHCCs) in Jeddah, working under Ministry of Health. This study included Saudi adults (Male and female) above 18 years visiting different PHCCs. The sample size was calculated according to available/previous prevalence of junk food consumption among adults in Saudi Arabia (from study conducted in Riyadh [8]) using Epi Tools Sample size calculator. According to this study, the prevalence of those who eat fast food at least every week is 60%. Using these figures, keeping the level of significance at 95% and desired precision at 0.5 the calculated sample size was 369. Multi-stage sampling technique was adopted. The PHCCs were stratified according to administrative distribution of Jeddah city (North, South, West, East and Middle Sectors). One PHCC was selected from each sector randomly using lottery method. Systematic sampling of the participants was done according to the calculated sample size (80 individuals from each center).

Data was collected in a face to face interview using a valid descriptive questionnaire with slight modification which was obtained from the previous study conducted in Saudi Arabia [8]. Data analysis was done on SPSS version 22 (IBM SPSS Statistics for Windows, Version 22.0. Armonk, NY: IBM Corp). Chi-square was used to analyze categorical variables. Variables that were significantly associated with junk food consumption in the chi-square analyses were included as covariates in the multivariate regression models (using a backward stepwise [likelihood Ratio] method) to evaluate the predictors for increased JF consumption. In logistic regression individuals consuming JF were referent group, and were compared via odds ratio to individuals who did not claim to be eating JF or were not regular JF consumers. To generate the best fit model, 16 independent variables have been entered and a total of 15 steps have been run.

All Saudi adults aged 18 and above, coming to selected PHCCs whether having disease or attendants and willing to participate were included in this study. However, non-Saudi coming to the PHCCs and who were not falling in the age group mentioned earlier were excluded. Official permission was taken from Ministry of Health and Directorate of Health Affairs Jeddah Public Health Management for data collection (H-02-J-002-00762). Verbal consent was taken from the participants after explaining the aim of this study and confidentiality of data was ensured.

## Results

The mean age of the study population was 33.69 ± 12.29 years. Of the 400 study participants, 36.5% (n = 146) were males and 63.5% (n = 254) were females. JF consumption was prevalent among all ages, both genders, belonging to different education level and occupational status (Table 1). Around 86.5% of the total sample reported consuming junk food (Table 1).

**Table 1:** Demographic and socioeconomic characteristics of study sample by junk food intake status.

Variable		Total n = 400 (%)	Individuals having junk food	p-value
Gender	Male	146 (36.5)	125 (85.6)	0.647
	Female	254 (63.5)	222 (87.4)	
Educational status	Secondary school education or lower	125 (31.3)	100 (80.0)	0.007
	College education or higher	275 (68.8)	247 (89.8)	
Occupation	Student	94 (24.0)	92 (95.8)	<0.001
	Unemployed	60 (15)	50 (83.3)	
	Employed	151 (37.8)	136 (90.1)	
	House wife	71 (17.8)	59 (83.1)	
	Retired	22 (5.5)	10 (45.5)	
Marital status	Single	135 (33.8)	127 (94.1)	<0.001
	Married	246 (61.5)	209 (85.0)	
	Divorced	16 (4.0)	10 (62.5)	
	Widow	3 (0.75)	1 (33.3)	
Living arrangements	Living alone	27 (6.8)	23 (85.2)	0.757
	Living with family	369 (92.3)	321 (87.0)	
	Living with friends	4 (1.0)	3 (75.0)	

**Figure 1:**
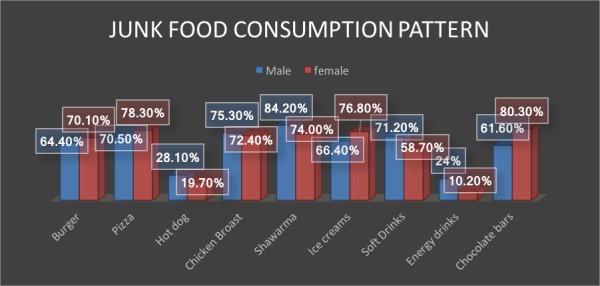
Gender differences in type of junk food.

A comparison of different forms of junk food preferences by males and females is given in Figure 1. Gender difference in the food preferences was less. Mostly burgers, pizzas, chicken broast were preferred food choices for both males and females. Major difference was noted in the consumption of hot dogs, shawarma and energy drinks, which were mostly liked by males however ice creams and chocolate bars, etc. were being utilized more by the females (Figure 1).

Females mostly eat junk food once or twice a week. However, a significant difference (p = 0.015) was noted in eating more than four to five times a week; males (17.8%) eating JF more than females (7.9%). Men prefer eating out over the weekends (38.4%) as compared to females (26.4%). Around 33.6% males were in favor of eating out from a local restaurant as they want to encourage local products (p = 0.029). Other gender differences are given in Table 2. Males usually prefer medium to large serving size (66.5%) however females prefer small serving size (56.3%) (Table 2).

**Table 2:** Gender differences regarding the use of junk food restaurants (n = 400).

Variables		Male, n = 146 (%)	Female, n = 254 (%)	p-value
How often do you eat junk food	Once or twice a week	57 (39.0)	126 (49.6)	0.015
	2-5 times/week	52 (35.6)	92 (36.2)	
	More than 4-5 times/week	26 (17.8)	20 (7.9)	
	Do not eat/not regular	11 (7.5)	16 (6.3)	
When do you usually consume JF	No specific time	68 (46.6)	155 (61.0)	0.017
	Beginning of week	5 (3.4)	2 (0.8)	
	Middle	6 (4.1)	14 (5.5)	
	Weekend	56 (38.4)	67 (26.4)	
	Do not eat/not regular	11 (7.5)	16 (6.3)	
From where do you usually buy JF	Local	49 (33.6)	32 (12.6)	<0.001
	International	20 (13.7)	56 (22.0)	
	Both	66 (45.2)	150 (59.1)	
	Do not eat/not regular	11 (7.5)	16 (6.3)	
Why do you prefer local places	Cheap	48 (32.9)	74 (29.1)	0.029
	Delicious	57 (39.0)	134 (52.8)	
	Encourage national products	30 (20.5)	30 (11.8)	
	Do not eat/not regular	11 (7.5)	16 (6.3)	
Why do you prefer international chains	Delicious	94 (64.4)	173 (68.1)	0.866
	Better service and standards	29 (19.9)	44 (17.3)	
	Original source	12 (8.2)	21 (8.3)	
	Do not eat/not regular	11 (7.5)	16 (6.3)	

**Figure 2:**
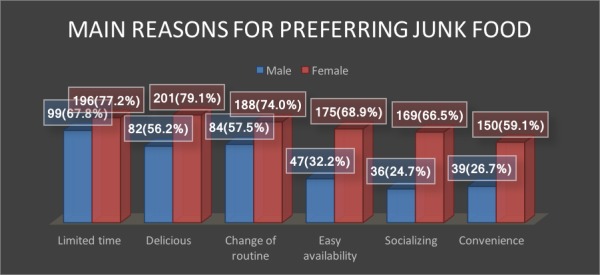
Main reasons for junk food preferences.

Bivariate analysis using chi-square shows an increased consumption of junk food depending on the emotional state. Increase in the junk food intake was noticed when the individuals were happy (p =< 0.001), angry or under stress (p =< 0.001). Unavailability of ample time, inspired by taste along with change of routine for majority (both males and females) were few main reasons for preferring junk food (Figure 2). Convenience and easy availability while hanging out and socializing with friends and family were also identified as major contributors for an increased junk food consumption especially among females (Figure 2).

Multiple logistic regression fit model for identifying more junk food consumption included only four independent variables (education, unavailability of time, change of daily routine and for scrumptious food) after controlling for age and gender, which are all significant. Table 3 indicates that the risk of JF consumption was two times increased among participants who were educated (OR = 2.47, 95% CI: 0.178-0.919, p = 0.031), and around four times in individuals who had limited time (OR = 3.81, 95% CI: 0.116-0.592, p = 0.001). Other results of regression analysis for individuals who wanted to change the routine and those who considered JF more delicious are given in Table 3.

**Table 3:** Multivariate regression analysis predicting factors for junk food consumption. B: Correlation coefficient; SE: Standard error; Wald: Wald Chi Square; Sig: Significant (p-value); Exp (B): Exponentiation B (Odds ratio); CI: Confidence interval.

Variables	B	SE	Wald	Sig	Exp (B)	95% CI	
						Lower	Upper
Education	-0.904	0.418	4.676	0.031	0.405	0.178	0.919
Limited time available	-1.341	0.416	10.372	0.001	0.262	0.116	0.592
Change of routine	-2.034	0.453	20.164	<0.001	0.131	0.054	0.318
Delicious taste	-2.401	0.490	23.968	<0.001	0.091	0.035	0.237
Constant	4.403	0.454	93.967	<0.001	81.729		

After adjusting for age and gender, variables (marital status, occupation, when angry, happy, under stress, delicious taste, attractive advertisements, easy availability with convenience, price and nutritional value) were excluded after logistic regression analysis (using a backward stepwise method).

## Discussion

This study highlighted the high prevalence of JF consumption among Saudi adults. A dramatic lifestyle change is noticed in Saudi population over the last few decades [8]. This change is not only in the form of sedentary lifestyle but also in the dietary patterns. This energy dense and sugar-sweetened modern day dietary pattern is at the expense of nutrient dense food [16,17]. A number of studies are highlighting these increasing trends of junk food consumption worldwide [12,15,17]. The trend of eating outside the home is becoming a regular component not only of the Western world, but the studies conducted in the developing countries also show an increase in it [7,16].

A study conducted in 2011 estimated that approximately 80% of Michigan adults aged 18 to 54 years went to fast-food restaurants at least once per month and 28% consumed fast food regularly (i.e., ≥2/week). The same study reported that fast food is consumed frequently in the United States and that prevalence of fast-food consumption has increased significantly in the past few decades [18]. Junk food consumption of more than two times per week (36%) by our female study participants was much more than 25% reported by Al-Hazzaa, et al. [19]. However, fast food consumption frequency (77%) of one to two times per week by our study participants was almost similar to study conducted in Riyadh where it was about 75% [20]. A study conducted in the United Kingdom indicated 58% adult residents using JF at least once a week [21]. Similarly, 28% Australian population is consuming take away fast food meals at least twice a week [22]. Another study conducted in Brazilian students showed that around 70% students eat JF four times or more per week [23]. A study conducted in 2010 in Riyadh on 127 adolescent Saudi girls (13-18 years) and 69 young adult Saudi girls (19-29 years) showed a vast majority of the participants (95.4%) eating out in restaurants. It was consumed once per week by 52.8% of adolescent girls and 60.9% of young adult girls [8]. A regional study in Kuwait in 2011 reported that fast food intake among Arab adults is about 92%. The weekly frequency intake of fast food was higher among men than women [24].

Different emotional factors also have an effect on the junk food consumption frequency. Studies show that fast food consumption increases when an individual is under stress. Similarly, it also increases when one is feeling positive and happy; the latter being a major factor for increased fast food consumption [16,17]. Similar findings were reported in our study participants too.

Burger and pizza was the most popular choice in the American college students [25]. Carbonated soft drinks have been reported to be the most frequently ordered beverage with food not only in America [25] but in other parts of Saudi Arabia also [19]. This current study findings replicate the findings of these two studies. Serving size of medium to large by men and small by the females was highlighted in our study. Almost similar findings were reported by Nora, et al. [8] and Driskell, et al. [25].

Although there was no specific time for consuming fast food, however, males (38.4%) were utilizing weekend more than females (26.4%). This could be due to the fact that men hanging out with friends over the weekend is common in our society. Our findings were not in accordance with the study conducted by Nora, et al. [8] where females mostly go out on weekends.

Study conducted in the USA [25] demonstrated limited available time as a main reason for preferring junk food; similar findings were revealed in our study too. This limited time is due to the modern day fast life style. Delicious taste is the second commonest reason for preferring junk food. Females mainly prefer JF because of its easy availability and convenience. Recent rapid mushrooming of outlets with free home delivery may be the possible reason. Similarly, a study conducted on Saudi young females showed delicious taste followed by convenience as the main reasons for increased junk food consumption [8].

Deliciousness of the local food was a common factor for attracting a lot of study subjects, similar to study findings of Nora, et al. [8]. Although the junk food consumption in both the genders was almost similar, but males prefer eating from a local brand more as compared to females. Reason identified for this preference was to encourage local brands. Females prefer international brands more because of its delicious taste and better services and standards. Brand names and attractive advertisements were not much an influence on our study participants as compared to Australian and American youth [26,27].

Regression analysis showed educated people going for more junk food. It may be because they are earning better than the less educated. People having routine home food the whole week want a change; therefore, they opt for junk food. Similar findings were noticed in studies conducted in other Saudi cities, Australia and the USA [8,19,26,27].

The main limitation of our study was not calculating body mass index (BMI) and not asking about their knowledge regarding harmful health effects of overconsumption of junk food products. Despite these limitations, the quantitative design gives a deep insight into factors influencing junk food consumption among Saudi adults.

## Conclusions

The study findings provided evidence on the high prevalence of junk food consumption among Saudi adults. The focus of our findings included individual’s preferences, consumption rates and identification of factors responsible for increased junk food utilization. In summary, junk food has influenced the dietary patterns of Saudi adults and this trend is likely to rise. This growing widespread use of junk food is of concern which may cause obesity-related NCDs.

To achieve a healthy life, JF consumption should be limited. To improve the dietary habits and food choices of Saudi population, community-based nutrition-related educational interventions should be conducted.
